# Discovery of a novel Nrf2 inhibitor that induces apoptosis of human acute myeloid leukemia cells

**DOI:** 10.18632/oncotarget.13825

**Published:** 2016-12-09

**Authors:** JinFeng Zhang, Le Su, Qing Ye, ShangLi Zhang, HsiangFu Kung, Fan Jiang, GuoSheng Jiang, JunYing Miao, BaoXiang Zhao

**Affiliations:** ^1^ Shandong Provincial Key Laboratory of Animal Cells and Developmental Biology, School of Life Science, Shandong University, Jinan 250100, China; ^2^ School of Municipal and Environmental Engineering, Shandong Jianzhu University, Jinan 250101, China; ^3^ Key Laboratory of Cardiovascular Remodeling and Function Research, Chinese Ministry of Education and Chinese Ministry of Health, Qilu Hospital, Shandong University, Jinan, 250012, China; ^4^ Institute of Pathology and Southwest Cancer Center, Third Military Medical University, Chongqing, 400038, China; ^5^ Key Medical Laboratory for Tumor Immunology and Traditional Chinese Medicine Immunology, Key Laboratory for Rare and Uncommon Diseases of Shandong, Institute of Basic Medicine, Shandong Academy of Medical Sciences, Jinan 250062, China; ^6^ Institute of Organic Chemistry, School of Chemistry and Chemical Engineering, Shandong University, Jinan 250100, China

**Keywords:** pyrazolyl hydroxamic acid derivatives, Nrf2 inhibitors, acute myeloid leukemia, apoptosis, Bcl-2

## Abstract

Nuclear factor-erythroid 2-related factor 2 (Nrf2) is persistently activated in many human tumors including acute myeloid leukemia (AML). Therefore, inhibition of Nrf2 activity may be a promising target in leukemia therapy. Here, we used an antioxidant response element-luciferase reporter system to identify a novel pyrazolyl hydroxamic acid derivative, 1-(4-(tert-Butyl)benzyl)-3-(4-chlorophenyl)-N-hydroxy-1H pyrazole-5-carboxamide (4f), that inhibited Nrf2 activity. 4f had a profound growth-inhibitory effect on three AML cell lines, THP-1, HL-60 and U937, and a similar anti-growth effect in a chick embryo model. Moreover, flow cytometry of AML cells revealed increased apoptosis with 4f (10 μM) treatment for 48 h. The protein levels of cleaved caspase-3 and cleaved poly (ADP-ribose) polymerase were enhanced in all three AML cell types. Furthermore, Nrf2 protein level was downregulated by 4f. Upregulation of Nrf2 by tert-butylhydroquinone (tBHQ) or Nrf2 overexpression could ameliorate 4f-induced growth inhibition and apoptosis. Treatment with 4f reduced both B-cell lymphoma-2 (Bcl-2) expression and Bcl-2/Bcl-2–associated X protein (Bax) ratio, which indicated that 4f induced apoptosis, at least in part, via mitochondrial-dependent signaling. Therefore, as an Nrf2 inhibitor, the pyrazolyl hydroxamic acid derivative 4f may be a promising agent in AML therapy.

## INTRODUCTION

Acute myeloid leukemia (AML) is the most common leukemia occurring in adults. The disease is due to abnormal differentiation of hematopoietic stem cells and is characterized by the accumulation of abnormal blasts in bone marrow [1]. From global gene profiling, approximately 50% of adults with AML feature chromosomal abnormalities, contributing to leukemia pathogenesis and heterogeneity [2, 3]. Some promising agents such as gemtuzumab ozogamicin (GO) [4], sorafenib [5] and AMG-330 [6] have been approved in preclinical and clinical trials. However, treatment-related mortality and drug resistance are the main obstacles that restrict their application. Therefore, new therapies and promising drug candidates are needed to overcome the inadequacy of traditional cytotoxic agents.

Nuclear factor-erythroid 2-related factor 2 (NFE2L2, also called Nrf2) is a ubiquitously expressed transcriptional factor that belongs to the cap’n’collar family of basic leucine-zipper (b-ZIP) proteins [7]. The Kelch-like ECH-associated protein 1 (Keap1)-Nrf2 system represents a major regulatory mechanism and controls adaptive responses to multiple stressors. Under normal homeostatic circumstances, Nrf2 binds with Keap1 for degradation to maintain a low level [8]. In response to reactive oxygen species (ROS) or electrophilic stress, Nrf2 dissociates with Keap1 and binds to antioxidant response element (ARE) localized in the promoter region, thereby inducing the expression of a wide array of genes [9] including heme oxygenase-1 (HO-1) [10], NAD(P)H:quinone oxidoreductase 1 [11], catalytic subunit of glutamate cysteine ligase (GCLC) [12] and multi-drug resistance gene family [13]. The precise mechanism of Nrf2 in tumorigenesis has been actively investigated *in vitro* and *in vivo* because of the pivotal role of Nrf2 as a defense mechanism against various cellular stressors in cancer cells [14–16]. Increasing evidence reveals that highly constitutive activation of Nrf2 is associated with increased risk of various human tumors [17, 18]. Nrf2 siRNA knockdown or inhibition of Nrf2 activity by some chemicals renders cancer cells susceptible to apoptosis [19, 20]. To date, several Nrf2 inhibitors, such as all-trans retinoic acid, other retinoic acid receptor α agonists [21], luteolin [22] and brusatol [23], have been identified. Therefore, the discovery and development of more Nrf2 inhibitors would be an attractive therapeutic strategy to improve AML therapy.

In this work, we used an ARE-luciferase reporter approach to screen a series of pyrazolyl hydroxamic acid derivatives and identified a novel compound, 1-(4-(tert-Butyl)benzyl)-3-(4-chlorophenyl)-N-hydroxy-1H pyrazole-5-carboxamide (4f), that inhibited Nrf2 activity, for an anti-growth effect on AML cells.

## RESULTS

### Effect of the pyrazolyl hydroxamic acid derivatives (4a-4l) on Nrf2 activity

**Figure 1:**
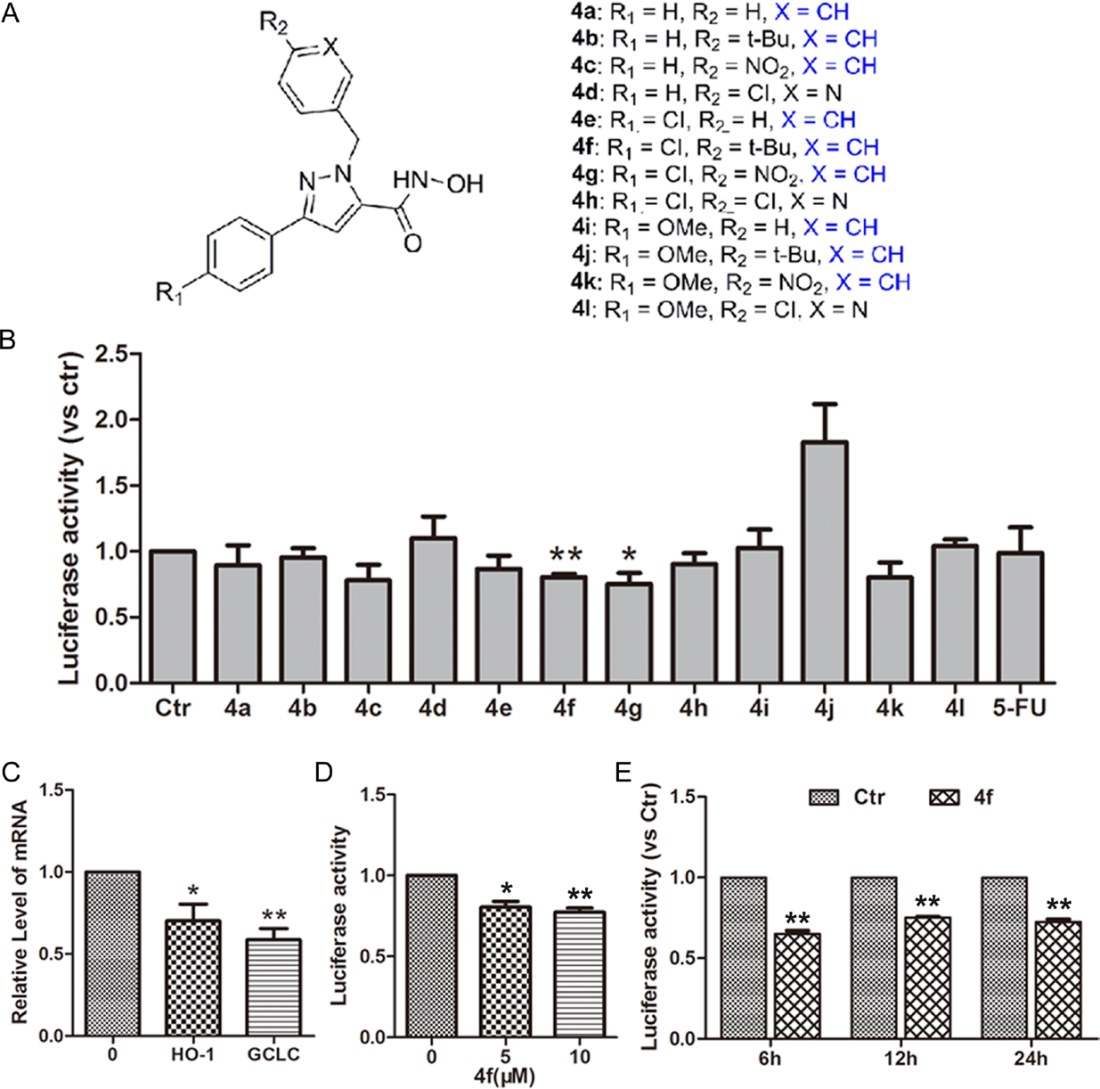
Effect of pyrazolyl hydroxamic acid derivatives (4a-4l) on Nrf2 activity (**A**) Chemical structures of pyrazolyl hydroxamic acids (4a-4l). (**B**) HeLa cells stably transfected with an ARE-luciferase reporter gene were incubated with compounds 4a-4l at 10 μM for 12 h. Luciferase activity was determined by luciferase assay, with control activity set to 1. (**C**) The expression of two target genes of Nrf2, HO-1 and GCLC, in treated cells was examined by RT-PCR. (**D**–**E**) The relative level of luciferase activity in HeLa cells incubated with 4f at 5 and 10 μM for 12 h or at 10 μM for 6, 12 and 24 h. Data are mean ± SEM. * *p* < 0.05, ** *p* < 0.01vs Ctr (untreated group), *n* = 3.

A cell-based Nrf2-luciferase system can be used to monitor an immediate response for high-throughput screening of Nrf2 modulators [24]. We used HeLa cells, which stably express functional ARE-driven reporter genes, to screen a series of pyrazolyl hydroxamic acid derivatives (4a-4l, Figure 1A). Luciferase activity was decreased with compound 4f or 4g (10 μM) incubation for 12 h but was maintained in other treated groups (Figure 1B), which suggests that both 4f and 4g inhibited Nrf2-ARE signaling. To confirm the effect on Nrf2 inhibition, we examined the mRNA levels of *HO-1* and *GCLC*, two downstream target genes of Nrf2. As expected, mRNA levels of *HO-1* and *GCLC* were down-regulated with 4f (10 μM) treatment for 12 h (Figure 1C). Furthermore, both 5 and 10 μM 4f decreased luciferase activity at 12 h as compared with controls (Figure 1D). A similar effect was observed with 4f (10 μM) treatment for different times (Figure 1E). Therefore, the results revealed that compound 4f inhibited Nrf2 activation.

### Effect of compounds 4f and 4g on the growth of three AML cell types

**Figure 2:**
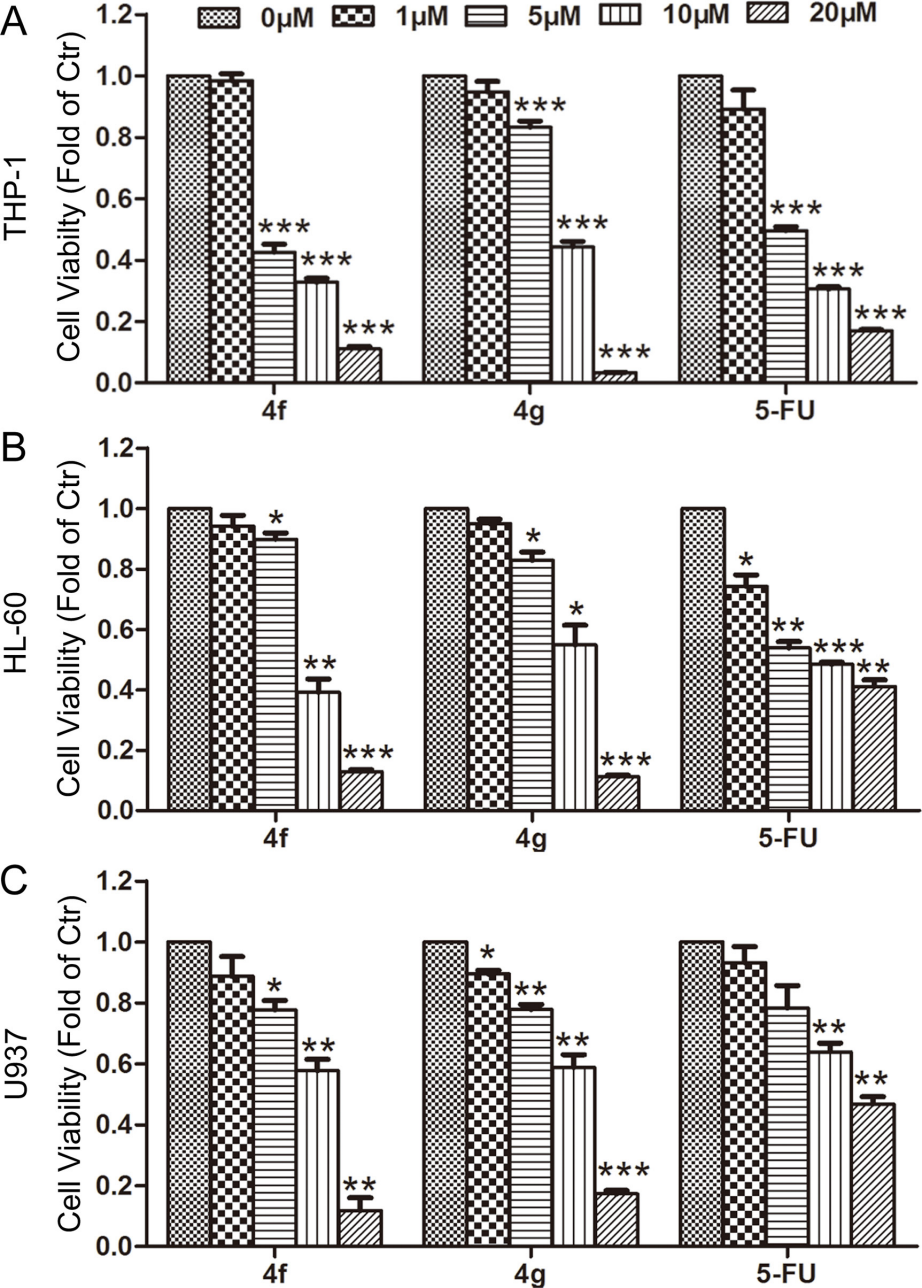
Effect of compounds 4f and 4g on the growth of three AML cell types THP-1, HL-60 and U937 cells were exposed to compound 4f or 4g at 1, 5, 10 and 20 μM for 48 h. Cell viability was measured by CCK-8 assay. 5-FU was used as a positive control. Viability of cells in controls was set to 1. Data are mean ± SEM. * *p* < 0.05, ** *p* < 0.01, *** *p* < 0.001 vs Ctr (untreated group), *n* = 3.

Next, we used CCK-8 assay to investigate the effect of 4f and 4g on the growth of three human AML cell lines, THP-1, HL-60 and U937. 4f or 4g inhibited growth of the three AML cell types at 5, 10 or 20 μM for 48 h (Figure 2). With increasing concentration, the cytotoxicity was enhanced accordingly for all tested cells. The growth-inhibitory ratio was even up to 80–90% at 20 μM. The half maximal inhibitory concentrations (IC_50_) for the three AML cell types ranged from 5 to 10 μM (Table 1). According to Nrf2 activity inhibition and cell viability, we chose 4f for further investigation.

**Table 1:** The IC_50_ values (μM, 48 h) of the compounds 4f and 4g in three acute myeloid leukemia cell types

Compound	4f	4g	5-FU
THP-1	5.33	8.23	5.18
HL-60	8.94	9.58	7.78
U937	8.98	9.79	18.10

### Compound 4f induces apoptosis of three AML cells *in vitro*

**Figure 3:**
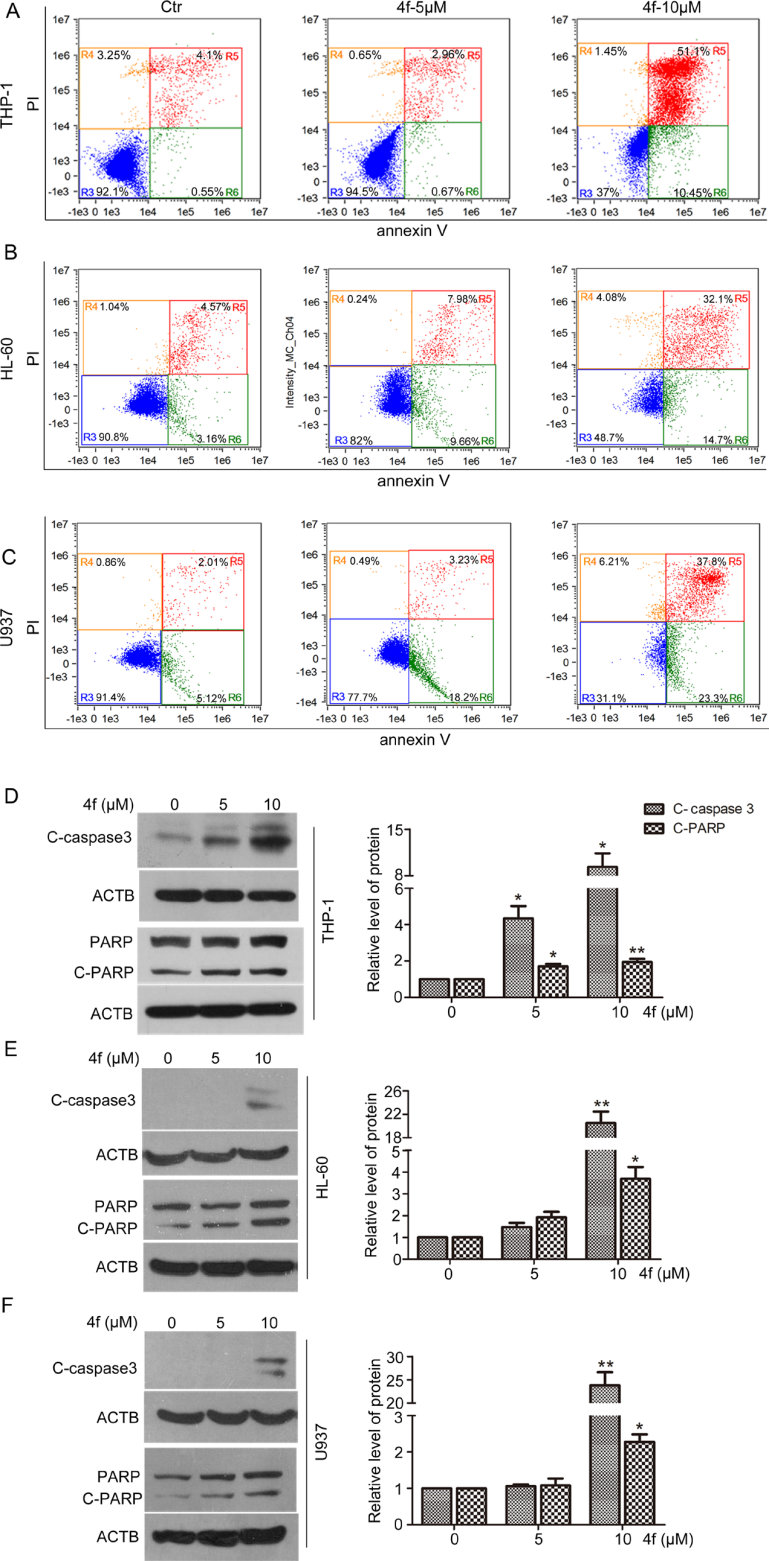
Compound 4f induces apoptosis in AML cells in vitro Cells were treated with the compound 4f (5 or 10 μM) for 48 h. (**A**–**C**) Apoptosis was determined by flow cytometry. (**D–F**) The protein levels of cleaved caspase 3 (C-caspase 3) and cleaved PARP (C-PARP) were detected by western blotting. β-actin (ACTB) was a loading control. The protein levels were normalized to ACTB. One representative experiment in 3 is shown. Data are mean ± SEM. * *p* < 0.05, ** *p* < 0.01vs Ctr (untreated group).

We performed flow cytometry to clarify whether apoptosis or necrosis was responsible for the 4f anti-leukemia effect. 4f at 10 μM markedly increased the proportion of apoptotic THP-1 cells (61.55%, Figure 3A). For HL-60 and U937 cells, approximately 46.8% of HL-60 cells and 61.1% of U937 cells were at early and late apoptosis stages. In 4f-treated AML cells (THP-1, HL-60 and U937), the percentages of early apoptotic cells were 10.45%, 14.7% and 23.3%, respectively. However, there are a few necrotic cells in HL-60 and U937 cells as compared with controls (Figure 3B–3C). Therefore, apoptosis may be the main mode of cell death in the three AML cell types.

Subsequently, we detected the activation of caspase-3 and poly (ADP-ribose) polymerase (PARP), the key proteins in apoptosis. The results showed a significant increase in level of cleaved caspase-3 (C-caspase 3) and PARP (C-PARP) with 10 μM 4f treatment (Figure 3D–3F). Thus, 4f triggered caspase-dependent apoptotic signaling in AML cells.

### Compound 4f inhibits tumor growth via apoptosis *in vivo*

**Figure 4:**
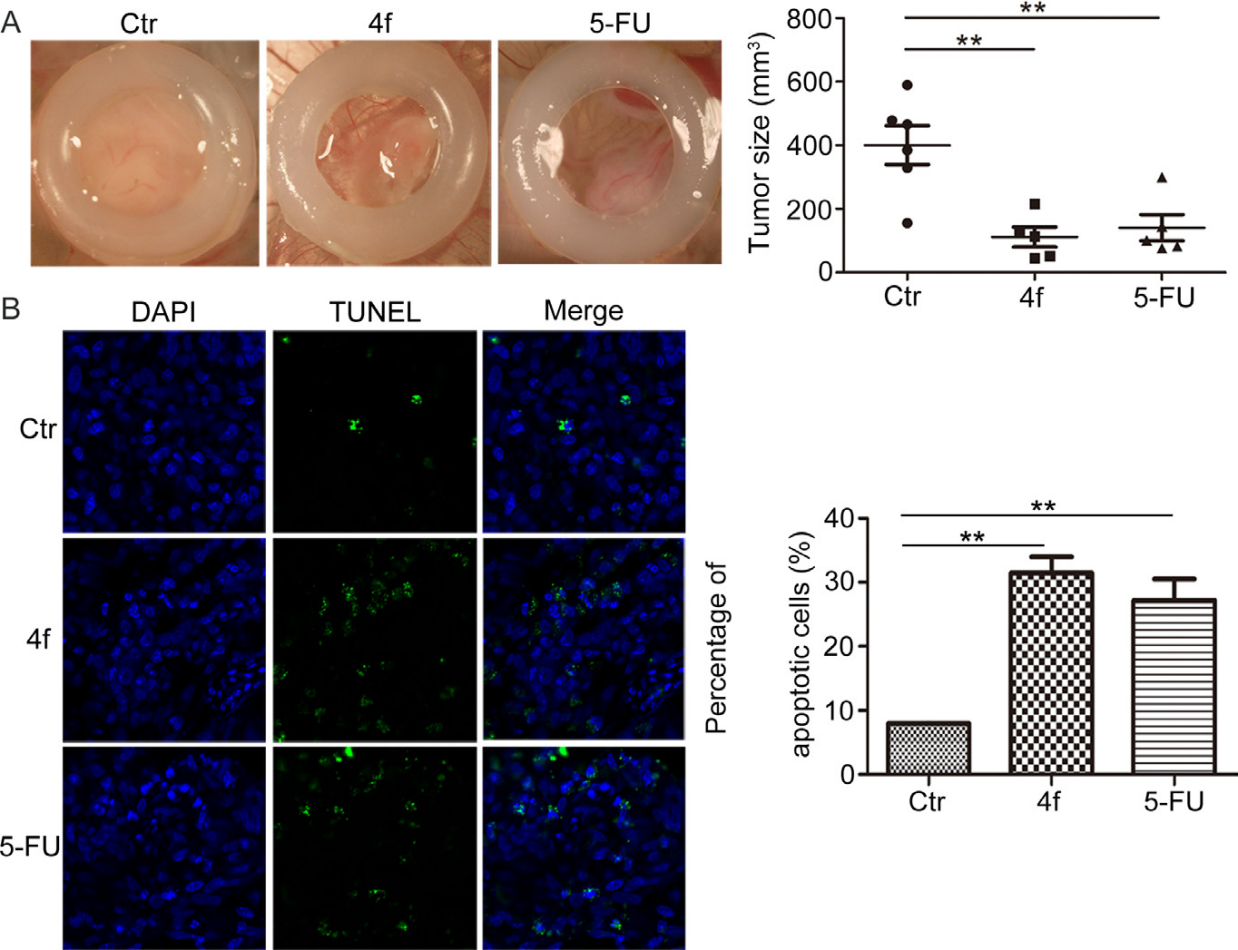
Compound 4f inhibits tumor growth via apoptosis in vivo (**A**) THP-1 cells were seeded onto the CAMs of chicken eggs to evaluate the tumor-growth-inhibitory effect of 4f *in vivo*. 5-FU was a positive control. The tumor size was calculated by densitometry analysis. Horizontal lines are means and whiskers are SEM. Data represent 1 sample. (**B**) TUNEL assay of sections of tumors (magnification 100×). Data are mean ± SEM. ** *p* < 0.01 vs Ctr.

The chorioallantoic membrane (CAM) of chick eggs is a well-established model for rapidly and inexpensively screening new anti-cancer drugs in many human cancers including blood malignancies [25, 26]. Therefore, we seeded cells on CAMs of chicken eggs and evaluated the tumor-growth-inhibitory effect of compound 4f treatment *in vivo*. The tumor size in CAMs was smaller with 4f than control treatment, with inhibitory rate approximately 40% (Figure 4A). Furthermore, to detect apoptotic cells, tumor sections were labeled by terminal deoxy-nucleotidyl transferase-mediated digoxigenin-dUTP nick-end labeling (TUNEL). 4f-treated tumor sections showed increased number of apoptotic cells as compared with controls (Figure 4B). Hence, compound 4f suppressed the growth of tumors in a chick embryo model, with apoptosis mainly accounting for reduced cell numbers.

### Nrf2 is decreased by compound 4f in three AML cells

**Figure 5:**
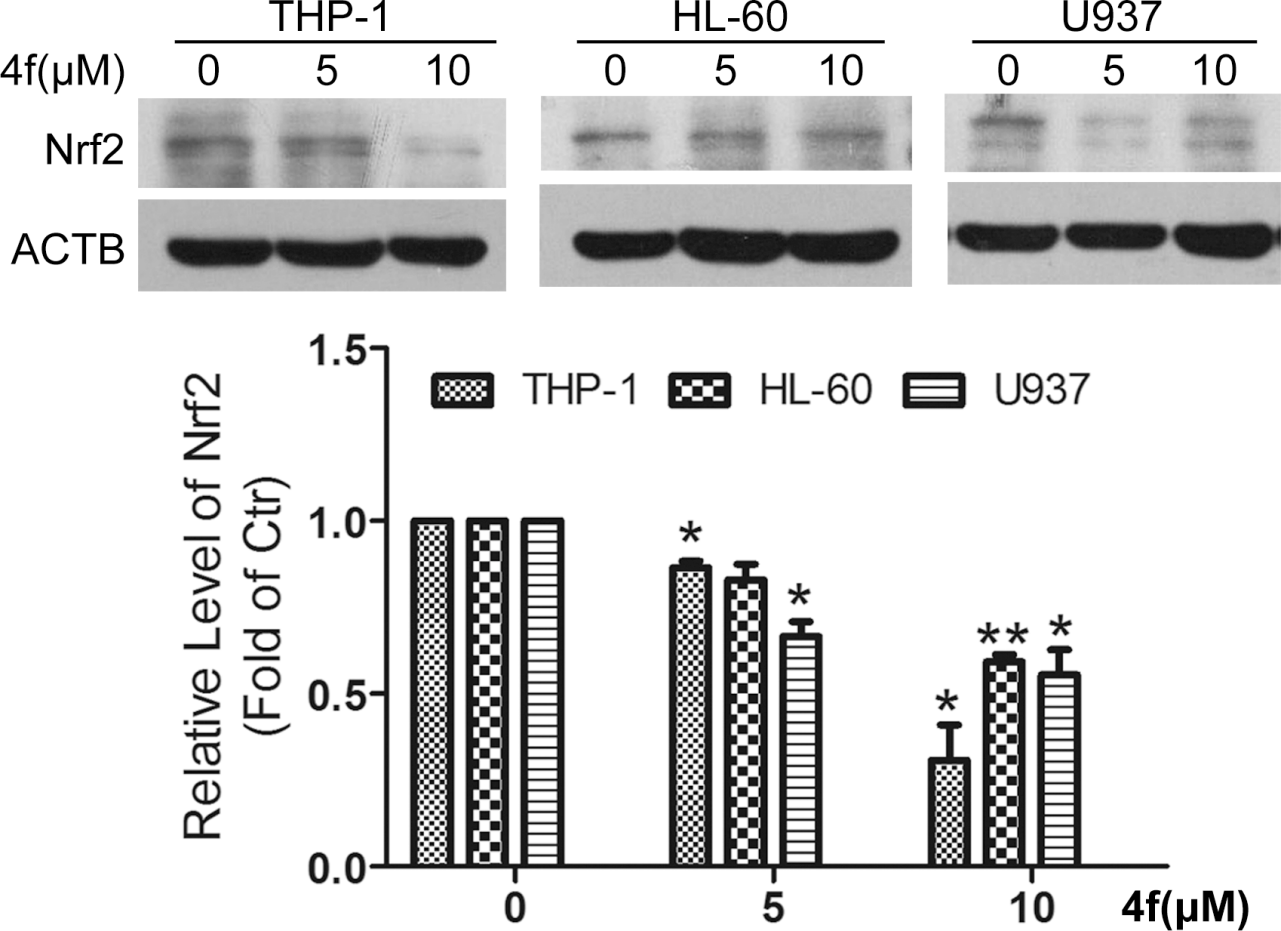
Nrf2 is decreased by compound 4f in three AML cells All three AML cells were exposed to 4f (5 and 10 μM) for 48 h, and then Nrf2 protein level was determined by western blot. The protein level of Nrf2 was normalized to ACTB (as a loading control). One representative experiment in 3 is shown. Data are mean ± SEM. * *p* < 0.05, ** *p* < 0.01 vs Ctr (untreated group), *n* = 3.

Despite compound 4f inhibited Nrf2 activity in ARE-luciferase reporter transfected HeLa cells, we examined whether Nrf2 protein level was altered in the three AML cell lines. As shown in Figure 5, 4f treatment led to the reduction of Nrf2 expression in THP-1 cells. Similarly, Nrf2 protein level was decreased in 4f-treated HL-60 and U937 cells.

### Upregulation of Nrf2 ameliorates 4f-induced growth inhibition and apoptosis

**Figure 6:**
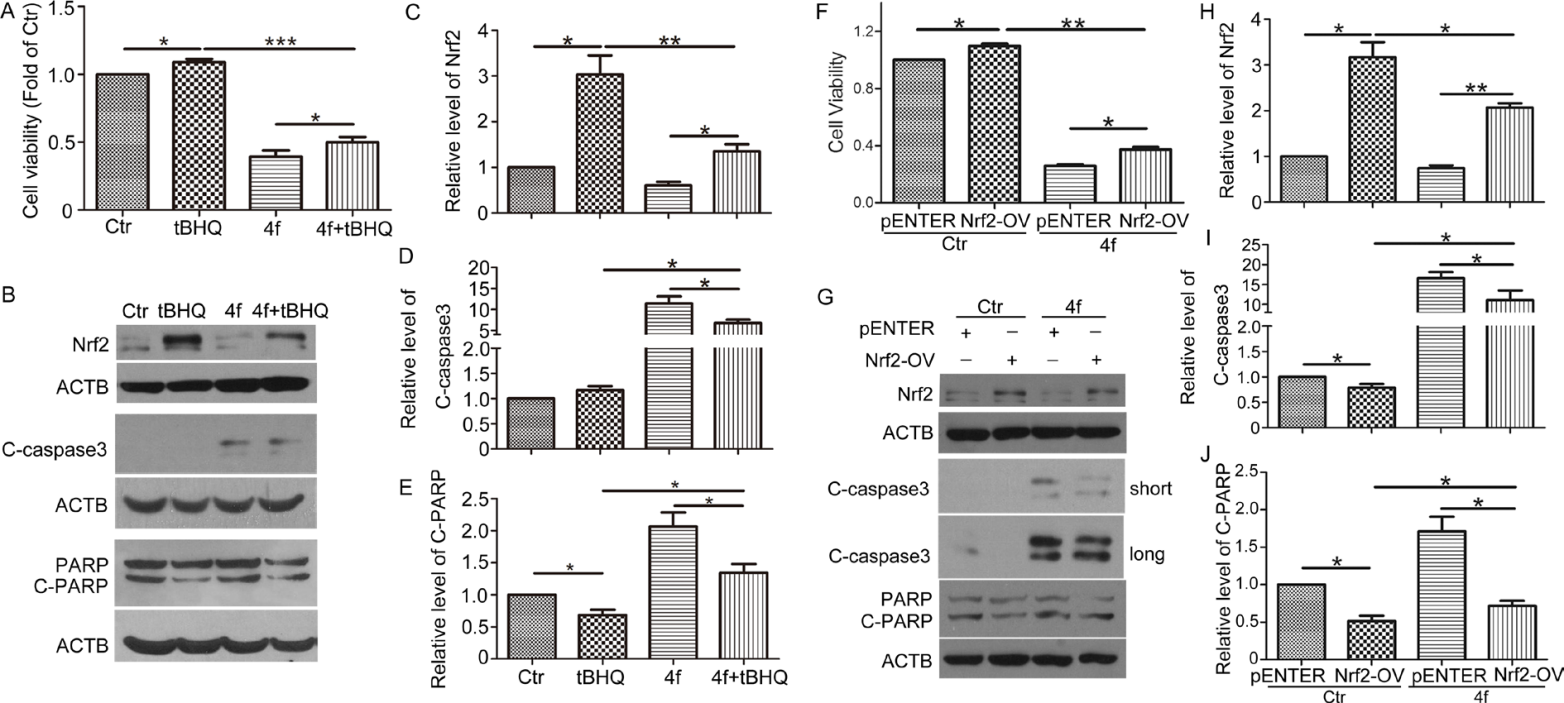
Upregulation of Nrf2 ameliorates 4f-induced growth inhibition and apoptosis After exposure to tBHQ (50 μM), 4f (10 μM) or tBHQ plus 4f for 48 h, THP-1 cell viability was measured by CCK8 assay (**A**). The protein levels of Nrf2, C-caspase 3 and C-PARP were detected by western blot analysis (**B**–**E**). After transfection with an Nrf2 expression plasmid (Nrf2-OV) or the corresponding control vector (pENTER) for 24 h, THP-1 cells were incubated with 4f for another 48 h. Cell viability was determined by CCK8 assay (**F**). Nrf2, C-caspase 3 and C-PARP were analyzed by western blot (**G**–**J**). Viability of cells in controls was set to 1. The protein levels were normalized to ACTB (a loading control). One representative experiment in 3 is shown. Data are mean ± SEM. * *p* < 0.05, ** *p* < 0.01, *** *p* < 0.001, *n* = 3.

Based on the above findings, we wondered whether growth inhibition and apoptosis induced by 4f were related to inhibiting Nrf2 expression. It has been documented that the antioxidant tBHQ is an Nrf2 activator promoting Nrf2 stability and inducing the expression of Nrf2-related genes [30–32]. Thus, we used tBHQ to clarify the role of Nrf2 in the effect of 4f. The addition of tBHQ increased THP-1 cell viability in the presence of 4f (Figure 6A). Meanwhile, Nrf2 protein level was upregulated, and C-caspase 3 and C-PARP level were downregulated (Figure 6B–6E). 4f plus tBHQ resulted in cell viability decreased as compared with tBHQ alone (Figure 6A), which was accompanied by decreased Nrf2 and enhanced expression of C-caspase 3 and C-PARP (Figure 6B–6E). Thus, activation of Nrf2 by tBHQ could ameliorate 4f-induced growth inhibition and apoptosis.

Furthermore, exogenous transfection of an Nrf2 plasmid in THP-1 cells was performed by electro-transfection followed by 4f treatment. Overexpression of Nrf2 significantly caused Nrf2 protein level upregulation (Supplementary Figure S1). Cell viability assay revealed that Nrf2 overexpression promoted cell growth and decreased the sensitivity to 4f treatment (Figure 6F). Both C-caspase 3 and C-PARP protein levels were decreased when compared with the vector groups (Figure 6G–6J). Treatment with 4f reversed cell viability, as well as the protein levels of Nrf2, C-caspase 3 and C-PARP in Nrf2-overexpressed cells. Therefore, these results demonstrate that growth inhibition and apoptosis by 4f are related to Nrf2 reduction.

### B-cell lymphoma-2 (Bcl-2) and Bcl-2–associated X protein (Bax) are involved in the apoptotic signaling induced by 4f

**Figure 7:**
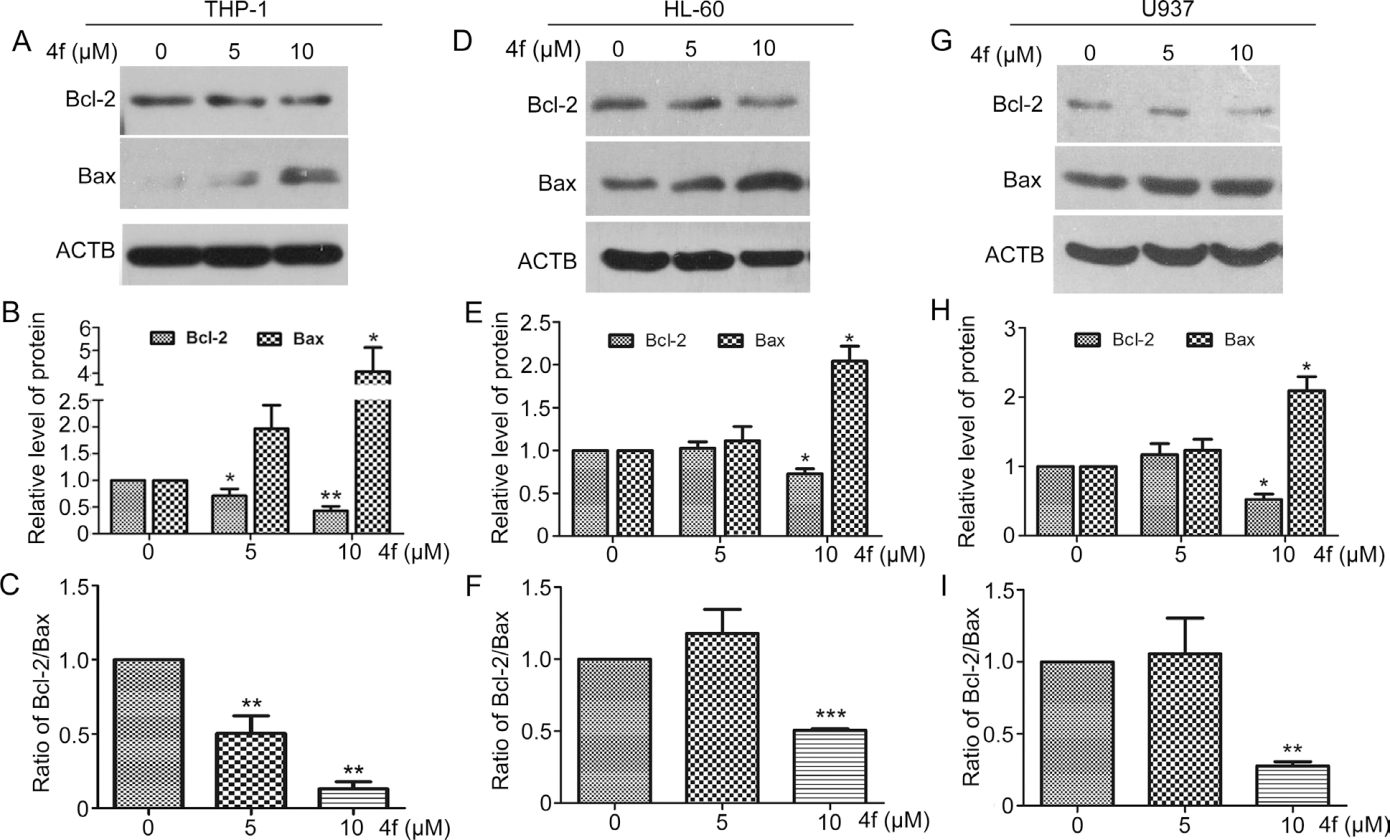
Bcl-2 and Bax are involved in the apoptotic signaling induced by 4f After incubation with 4f at 5 or 10 μM for 48 h, Bcl-2 and Bax protein levels were examined by western blot analysis (**A**–**C**) for THP-1 cells, (**D**–**F)** for HL-60 cells and (**G**–**I**) for U937 cells). One representative experiment in 3 is shown. ACTB was a loading control. Data are mean ± SEM. * *p* < 0.05, * * *p* < 0.01, *** *p* < 0.001 vs Ctr (untreated group).

To explore the mechanism by which compound 4f induced apoptosis, we examined Bcl-2 and Bax, two members of Bcl-2 family involved in mitochondria-dependent apoptotic signaling [27]. Bax protein level was increased and that of Bcl-2 protein level and ratio of Bcl-2/Bax were decreased when THP-1 cells were exposed to 4f at 5 and 10 μM for 48 h (Figure 7A–7C), with similar effects in the two other AML cell types (Figure 7D–7I). Thus, involvement of Bcl-2 and Bax in mitochondria-dependent apoptotic signaling may contribute to 4f-induced apoptotic cell death.

## DISCUSSION

In the present work, we identified a novel pyrazolyl hydroxamic acid derivative, 4f, with potential inhibition of Nrf2, which is persistently activated in many human tumors including AML. 4f showed effective anti-leukemia activity and triggered apoptosis via mitochondria-dependent signaling, which suggests its potential application in AML therapy.

In this study, a luciferase reporter system revealed that among pyrazolyl hydroxamic acid derivatives, compounds 4f and 4g inhibited Nrf2 activity (Figure 1B), which was further confirmed by *HO-1* and *GCLC* mRNA downregulation (Figure 1C). Moreover, we found a decreased Nrf2 protein level in 4f-treated AML cells (Figure 5). In contrast to isoniazid, with high ARE activity inhibition (IC_50_ 10 mM) [28], the concentration of 4f or 4g in suppressing Nrf2 is smaller. Further study revealed that the two compounds at higher concentration remarkably inhibited growth of THP-1, HL-60 and U937 AML cells (Figure 2). 4f had better anti-growth effect than 4g, and the IC_50_ value for 4f was smaller in THP-1 than HL-60 and U937 cells (Table 1). In addition, Nrf2 activation by tBHQ or Nrf2 overexpression could reverse 4f-induced THP-1 cell viability reduction and apoptosis (Figure 6A). Therefore, compound 4f inhibits AML cell growth, which is associated with Nrf2 suppression. Consistent with our results, a recent study showed that silencing of Nrf2 in THP-1 cells sensitized the cells to cytotoxicity by multiple agents [29]. Additionally, Nrf2 activation was found to protect AML cells against As_2_O_3_ or bortezomib-induced apoptosis [29, 30]. The evidence has implied more effective growth inhibition of cancer cells when combining the compounds with Nrf2 inhibitors, such as 4f.

ROS levels are higher in cancer cells than normal cells. Human tumors show persistent activation of an Nrf2-mediated response in defense against oxidative stress [18, 31]. Nrf2 activation and ROS levels feature a negative loop, in that persistent Nrf2 inhibition leads to intracellular ROS accumulation. Excessive ROS would lead to irreversible biomolecule oxidization, mitochondrial proper function impairment and cell death [32]. In the current work, there was a significant increase in apoptotic cells after exposure to 4f in a dose-dependent manner, which was accompanied by C-caspase 3 and C-PARP protein levels upregulated (Figure 3). Additionally, 4f-treated tumor sections showed increased number of apoptotic cells (Figure 4B). It should be noted that the proportion of late apoptotic and necrotic cells was higher than that of early apoptotic cells (Figure 3A–3C), suggesting that it may be better to use 4f at a lower dose in AML treatment. In a recent study by our laboratory, apoptosis was not triggered in 4f-treated A549 cells [33], which implies that the difference in cell death induced by 4f largely depends on cell type. We also found that cell cycle progression was arrested at the S phase by 4f in THP-1, HL-60 and U937 cells (Supplementary Figure S2). Hence, apoptosis and cell cycle arrest are mainly responsible for the growth-inhibitory effect of 4f in the three AML cells.

The CAM system has long been used to study the growth and spread of mammalian tumors [25, 34], as well as the angiogenesis and anti-angiogenesis potential [35, 36]. In this study, 4f not only suppressed the growth of THP-1 xenografts, but also blocked blood vessel development in an *in vivo* gelatin sponge assay (Supplementary Figure S3A). In agreement with the results, 4f (1, 5, 10 and 20 μM) had a dose-dependent growth-inhibitory effect on human umbilical vein endothelial cells (HUVECs) and SV40 T-antigen immortalized murine endothelial cells (MS1) (Supplementary Figure S3B and S3C). Therefore, we preliminarily ascertained that compound 4f inhibited angiogenesis. However, the detailed mechanism(s) requires further studies.

The Bcl-2/Bax ratio is considered an indicator of mitochondrial permeability [37, 38]. Increased Bcl-2 inhibits cell apoptosis in response to many chemotherapeutic agents [39, 40]. Recently, a study pointed out that Nrf2 deletion was associated with mitochondrial permeability, dysfunction and increasing apoptosis in murine embryonic fibroblasts [41]. Here, we found both Bcl-2 protein level and Bcl-2/Bax ratio decreased during 4f-induced apoptosis (Figure 7), indicating that the normal function of mitochondria was disturbed. The direct relationship between Nrf2 and Bcl-2 expression was identified, in which Nrf2 directly bound to an ARE in the promoter region of *Bcl-2* gene to upregulate *Bcl-2* expression and increase the survival of cancer cells [14]. Consistent with this notion, our results illustrated an association between Nrf2 and Bcl-2 because both Nrf2 activity and *Bcl-2* mRNA expression were decreased with 4f treatment (Figure 1B–1E and Supplementary Figure S4). Therefore, Nrf2 inhibition by 4f may lead to apoptosis, at least in part, by mitochondrial-dependent signaling.

**Figure 8:**
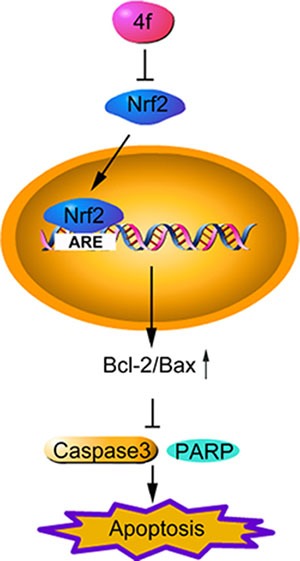
The schematic diagram of compound 4f-induced apoptosis by inhibiting Nrf2 in AML cells Compound 4f inhibits Nrf2 and its downstream gene expression, then triggers apoptosis via upregulating Bcl-2/Bax ratio and downregulating cleaved caspase 3 and cleaved PARP. Therefore, 4f has a profound anti-growth effect on three AML cell types (THP-1, HL-60 and U937).

Taken together, in the present work, we found that a novel Nrf2 inhibitor, 4f, had a profound growth-inhibitory effect on three AML cell types (THP-1, HL-60 and U937). After suppressing Nrf2 and Bcl-2 expression, 4f triggered apoptosis via mitochondrial-dependent apoptotic signaling (Figure 8). Therefore, as an Nrf2 inhibitor, 4f is a promising lead compound that may have application in advanced AML treatment. Nonetheless, how 4f inhibits Nrf2 remains for further investigation.

## MATERIALS AND METHODS

### Synthesis of pyrazolyl hydroxamic acids (4a-4l)

The detailed procedure for the synthesis and structures of pyrazolyl hydroxamic acids (4a-4l) (Figure 1A) were described in our previous study [33].

### Reagents

RPMI-1640 and DMEM (with high glucose) medium powder were from Gibco (Carlsbad, CA, USA). Fetal bovine serum (FBS) was from Hyclone (USA). The CCK-8 kit was from Dojindo Laboratories (Kumamoto, Japan). The FITC/Annexin V apoptosis detection kit with propidium iodide (640914) was from BioLegend (San Diego, CA, USA). Kits for TUNEL assay (*In Situ* Apoptotic Detection) (G3250) and luciferase assay (E1500) were from Promega (Madison, WI). TRIzol reagent (T9424) was from Sigma-Aldrich (St. Louis, MO). PrimeScript RT Master Mix (RR047A) was from Takara (Shiga, Japan). The QuantiTect SYBR Green PCR kit (204143) was from QIAGEN (Germany). Lysis buffer (P0013) and the bicinchonininc acid kit (BCA, P0011) were from Beyotime (Shanghai, China). Antibodies for PARP (9542S), cleaved caspase-3 (9661) and β-actin (A5441) were from Cell Signaling Technology (Danvers, MA, USA). Antibodies for Nrf2 (16396-1-AP), Bcl-2 (12789-1-AP) and Bax (50599-2-Ig) were from Proteintech (Chicago, IL, USA). Horseradish peroxidase (HRP)-conjugated secondary antibodies were from Santa Cruz Biotechnology (Santa Cruz, CA, USA). tBHQ (≥ 97.0%) was from Sigma-Aldrich. Dimethyl sulfoxide (DMSO) was from Beijing Solaris Science and Technology Company (Beijing, China). All compounds were dissolved in DMSO at 100 mM and diluted into the indicated concentrations in culture medium before use, and the final concentration of DMSO (v/v) was < 0.1%.

### Cell culture

Human acute monocytic leukemia cell lines THP-1, U937 and human promyelomonocytic leukemia cell line HL-60 were grown in RPMI-1640 medium containing 10% (v/v) FBS supplemented with streptomycin (100 μg/mL) and penicillin (100 U/mL). HeLa cells (human cervical cancer cell line) stably transfected Nrf2-responsive reporter plasmid (pGL4-3 × ARE) were a kind gift from Prof. Fan Jiang and were grown in DMEM media with high glucose. Cells were cultured at 37 °C in a humidified atmosphere of 5% CO_2_. Cells were plated in the indicated dishes for 24 h before treatment with compounds.

### Luciferase assay

HeLa cells stably expressing an Nrf2-responsive reporter plasmid (pGL4-3 × ARE) were seeded in 96-well plates at 4 × 10^4^/ml and cultured overnight, then incubated with the pyrazolyl hydroxamic acid derivatives at the indicated concentrations and times. After cell lysis buffer was added and collected, luciferase activity was examined by use of the luciferase assay system. The final luciferase activity was normalized to cell viability assessed by MTT assay.

### Cell viability assay

Cell viability was assessed by use of the CCK-8 kit according to the manufacturer's instructions. Briefly, THP-1, HL-60 and U937 cells were seeded in 96-well plates before exposure to compounds 4a-4l (0, 1, 5, 10 and 20 μM) or 5-fluorouracil (5-FU, as positive) for 48 h. WST-8 solution (10 μL) was added to plates for further incubation. At 2 h later, the absorbance at wavelength 450 nm was measured by using the Tecan Infinite 200 PRO microplate spectrophotometer (Tecan Austria GmbH). Cell viability was calculated and normalized to the value for control cells.

### Flow cytometry

After adherent and detached cells were harvested and washed with PBS twice, cells were suspended with binding buffer (100 μL) and stained with FITC/annexin V (8 μL) and PI (5 μL) for 30 min at 4 °C. Then, cells were suspended with binding buffer (50 μL) and examined by flow cytometry (Amnis ImageStream Marksims II, USA). Data were analyzed by using IDEAS image analysis software.

### Overexpression of Nrf2 in THP-1 cells

Cells were collected into the electroporation cuvettes (4 mm, Bio-Rad, USA) at a density of 1 × 10^7^/ml before transfected with a pENTER-Nrf2 expression plasmid or the corresponding control vector (Vigene Biosciences, USA). 36 h later, the transfection efficiency was determined by western blot.

### RT-PCR analysis

Cells were incubated with or without the compound 4f for the indicated times. Total RNA was isolated by the TRIzol reagent method and RNA content was determined by measuring OD_260_. cDNA was synthesized by using the PrimeScript RT reagent kit with gDNA Eraser. RT-PCR involved use of the QuantiTect SYBR Green PCR kit with 2 μL cDNA template, 10 μL of 2× SYBR Green, 0.4 μL forward primers, 0.4 μL reverse primers and 7.2 μL distilled water. RT-PCR cycling was at 95°C for 5 min, 95 °C 30 s, 56 °C 30 s and 72 °C 30 s for 40 cycles. Relative expression of target genes was analyzed by the 2^−ΔΔCt^ method and normalized to β-actin level. The primer sequences are in Supplementary Table S1.

### Western blot analysis

After cells were lysed in lysis buffer and protein concentrations were determined by BCA protein assay, equal amounts of whole protein lysates (20–50 μg) were separated by 12–15% SDS-PAGE and electrophoretically transferred to polyvinylidene fluoride membranes (Millipore, Schwalbach, Germany). The membranes were blocked with 5% non-fat dry milk in Tris buffer saline containing 0.5% Tween 20 (TBST) and incubated with primary antibodies at 4 °C overnight, then HRP-conjugated secondary antibodies for 1 h at room temperature before two additional washes with PBS. Proteins were detected by use of an ECL detection kit (Thermo Fisher Scientific, USA, 35060). Image J (US National Institutes of Health, USA) was used to quantify protein bands on X-ray film.

### Engraftment of THP-1 cells in chicken embryos

Fertile chicken eggs were incubated with turning at 37.5°C and 60% humidity. After a small aperture was cut in the eggshell, cells at (1–10) × 10^6^ were suspended in 20 μL RPMI 1640 medium and layered onto the large CAM of eggs at embryonic day 7 to 9 (E7–9). Egg shells were sealed with gas-permeable tape, then eggs were randomized to three groups and incubated for another 7 days with injection of PBS (negative control), 4f (50 μM) or 5-FU (positive group) every 48 h. Embryos were removed on E14–16 and killed by rapid decapitation. CAMs were separated from eggs, fixed with 4% paraformaldehyde for 30 min and photographed by use of a stereomicroscope (Japan). Data were quantified by using Image J. Tumor size was based on tumor volume calculation: length × width × width × 0.5.

### Terminal deoxynucleotidyl transferase-mediated dUTP nick-end labeling (TUNEL)

Excised tumor tissue was embedded in optimal cutting temperature (OCT) medium (Tissue-Tek, 4583) and cut into cryosections of 7 μm. Apoptotic cells in tumor sections were detected by TUNEL assay according to the manufacturer's instructions. Briefly, cryosections were fixed with 4% paraformaldehyde for 30 min and incubated with 0.2% Triton X-100 for 5 min, then with TUNEL reaction solution for 1 h at 37°C before SSC solution was added to terminate the reaction. After washes with PBS three times, sections were stained with DAPI for 5 min and observed by confocal microscopy (Carl Zeiss, Germany).

### Statistical analysis

All data were presented as mean ± SEM from least three independent experiments. Data analysis involved Student's *t* test with GraphPad Prism 5.0. Differences were considered statistically significant at *p* < 0.05.

## SUPPLEMENTARY MATERIALS FIGURES AND TABLES






