# Supplementary material for: Discovery of a novel Nrf2 inhibitor that induces apoptosis of human acute myeloid leukemia cells
Source: Oncotarget. 2016 Dec 9;8(5):7625–36. doi: 10.18632/oncotarget.13825 (PMC5352348; doi:10.18632/oncotarget.13825)
Supplement: Supplementary file 1 [file oncotarget-08-7625-s001.pdf]

Discovery of a novel Nrf2 inhibitor that induces apoptosis of human acute myeloid leukemia cells

Supplementary Materials

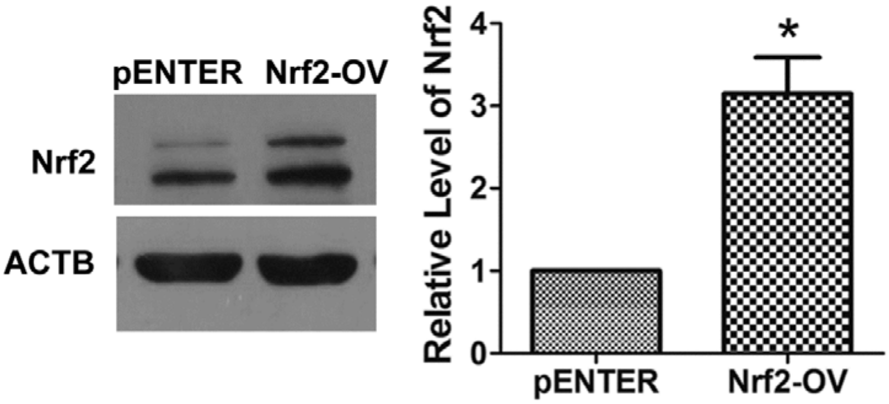

Supplementary Figure S1: Overexpression of Nrf2 increases Nrf2 protein level in THP-1 cells. THP-1 cells at a density of 1 × 10^7/ml were transfected with an Nrf2 expression plasmid (Nrf2-OV) or the corresponding control vector (pENTER) by electro-transfection (100 V for 25 ms). 36 h later, the transfection efficiency was determined by western blot. The detected band of Nrf2 is at ~90–110 KD. β-actin (ACTB) was a loading control. The protein levels were normalized to ACTB. One representative experiment in 3 is shown. Data are mean ± SEM. *$p < 0.05$, $n = 3$.

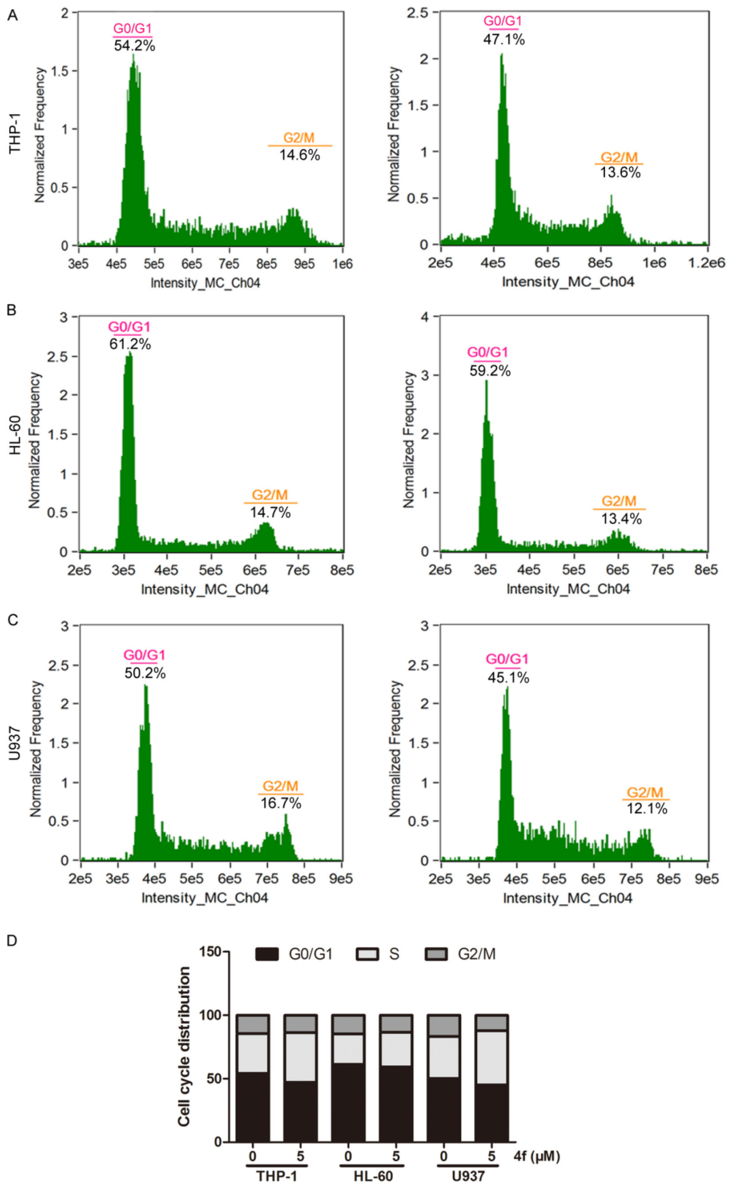

Supplementary Figure S2: Compound 4f blocks cell cycle progression in three AML cell types. Three AML cells types (THP-1 (**A**) HL-60 (**B**) and U937(**C**)) were treated with compound 4f at 5 μM for 24 h, and the cell cycle distribution was examined by flow cytometry. The distribution of cell cycle was shown in (**D**).

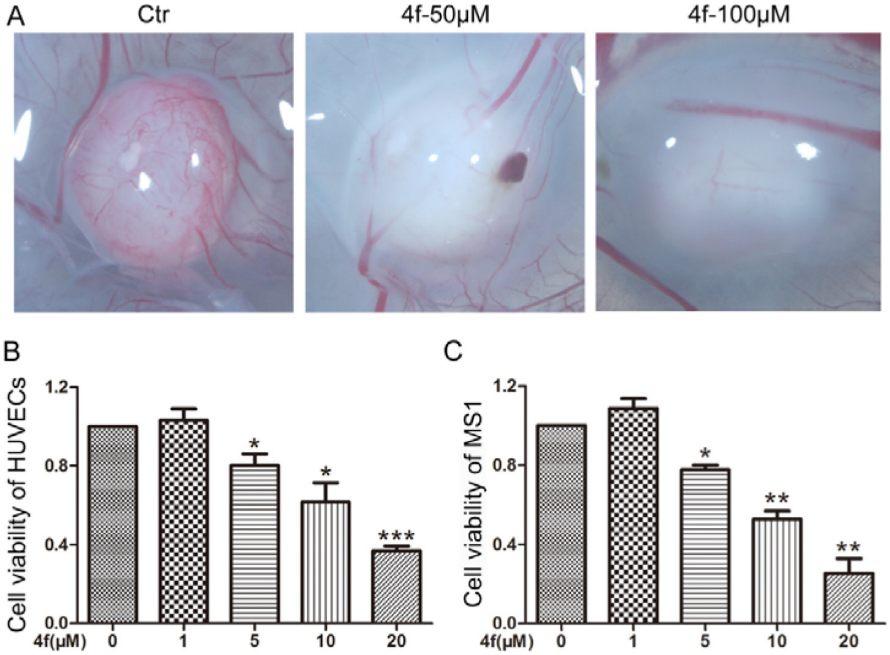

Supplementary Figure S3: Compound 4f inhibits blood-vessel development in chick embryos and endothelial-cell growth *in vitro*. (**A**) After 1 week of dosing, 4f inhibited blood vessel development on *in vivo* gelatin sponge assay in the CAM system. Data represent 1 sample. (**B–C**) sulforhodamine B assay was used to explore the effect on the growth of human umbilical vein endothelial cells (HUVECs) and SV40 T-antigen immortalized murine endothelial cells (MS1). Growth-inhibitory effect of 4f (at 1, 5, 10 and 20 μM for 48 h) on HUVECs and MS1 cells was shown. Data are mean ± SEM. *$p < 0.05$, **$p < 0.01$, ***$p < 0.001$, $n = 3$.

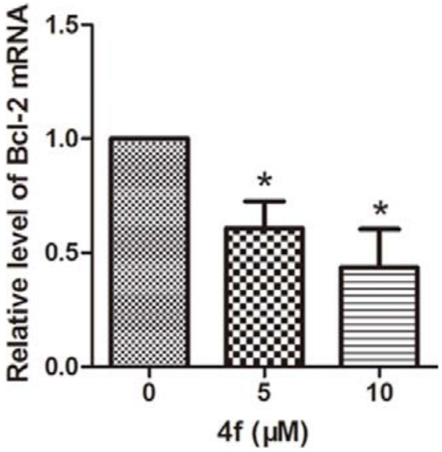

Supplementary Figure S4: Compound 4f downregulates Bcl-2 mRNA level in THP-1 cells. After THP-1cells were exposed to the compound 4f (5 and 10 μM) for 48 h, Bcl-2 mRNA level was analyzed by RT-PCR. Data are mean ± SEM. *$p < 0.05$ vs Ctr (untreated group), $n = 3$.

Supplementary Table S1: Primer sequences used in RT-PCR

HO-1	FP	5′-TGCGGTGCAGCTCTTCTG-3′
	RP	5′-GCAACCCGACAGCATGC-3′
GCLC	FP	5′-GGCGATGAGGTGGAATAC-3′
	RP	5′-AAAGGGTAGGATGGTTTGG-3′
Bcl-2	FP	5′-CGGGAGATGTCGCCCCTGGT-3′
	RP	5′-GCATGCTGGGGCCGTACAGT-3′
β-actin	FP	5′-GAAGTGTGACGTGGACATCC-3′
	RP	5′-CCGATCCACACGGAGTACTT-3′
