# Supplementary material for: Discovery of a novel Nrf2 inhibitor that induces apoptosis of human acute myeloid leukemia cells
Source: Oncotarget. 2016 Dec 9;8(5):7625–36. doi: 10.18632/oncotarget.13825 (PMC5352348; doi:10.18632/oncotarget.13825)
Supplement: Supplementary file 3 [file oncotarget-08-7625-s003.docx]

| **Supplementary Table S1: Primer sequences used in RT-PCR** | | |
| --- | --- | --- |
| HO-1 | FP | 5′-TGCGGTGCAGCTCTTCTG-3′ |
|  | RP | 5′-GCAACCCGACAGCATGC-3′ |
| GCLC | FP | 5′-GGCGATGAGGTGGAATAC-3′ |
|  | RP | 5′-AAAGGGTAGGATGGTTTGG-3′ |
| Bcl-2 | FP | 5′-CGGGAGATGTCGCCCCTGGT-3′ |
|  | RP | 5′-GCATGCTGGGGCCGTACAGT-3′ |
| β-actin | FP | 5′-GAAGTGTGACGTGGACATCC-3′ |
|  | RP | 5′-CCGATCCACACGGAGTACTT-3′ |
